# "The Hospital" Nursing Mirror

**Published:** 1898-10-29

**Authors:** 


### "The Hospital" Nursing Mirror.

Being the Nursing Section of "The Hospital."

[Contributions for this Section of "The Hospital" should be addressed to the Editor, The Hospital, 28 & 29, Southampton Street, Strand, London, W.C., and should have the word "Nursing" plainly written in left-hand top corner of the envelope.]

### News from the Nursing World.

### ROYAL ENGAGEMENTS.

H.R.H. the Princess Louise has promised to open the new wards at the West London Hospital, Hammersmith. New arrangements for the comfort of the nurses have just been completed, and a small adjacent house fitted up for their reception. We hear also that the Duchess of Albany has promised to perform the ceremony of opening the new nurses' home in connection with the Chelsea Hospital for Women, Fulham Road, at the end of January next.

### GERMAN RED CROSS MEDAL.

In deference to a wish of the Empress, the German Emperor has inaugurated a new medal for members of the Red Cross Society. It will be struck in gold, silver, and bronze, in order to designate three ranks of wearers, and will be known as the "Red Cross Medal."

### FLORAL BAZAAR AT GROSVENOR SQUARE.

Lady Percy very kindly lent her house at 28, Grosvenor Square, on Wednesday and Thursday afternoons for a floral bazaar in aid of the Plaistow Maternity Charity and Nurses' Home. She also provided much of the fine fruit and flowers for sale. The stalls were decorated by Miss Edith Webster, and the effect, achieved principally by chrysanthemums, palms, and coloured leaves, was charming. Selections of songs and music were given gratuitously by Miss Grainger Kerr, Miss Fennings, Miss Pulvermacher, Miss Heap, Mdlle. Marie Ernst (violin), Mr. Penderel Price, Mr. Frederick Keel, and others.

### A JEWISH CONVALESCENT HOME.

The well-known Baroness Hirsch, who has never lived in England, has recently purchased Tudor House, Hampstead Heath, as a convalescent home for poor London Jews. The cost of the house is £16,000, and the generous donor has given another £40,000 as an endowment. When the necessary alterations have been made accommodation will be afforded for 18 men and 18 women. This is the third convalescent home for Jews, there being one in London and one in Brighton. The rules regulating the dietary of the Israelites prevent their availing themselves of the ordinary homes, and it will be remembered that at the London Hospital there is a special kitchen set apart for the preparation of their food. This is a perfectly fair arrangement, as Jews subscribe liberally to such charities.

### ROYAL BRITISH NURSING ASSOCIATION.

The Royal British Nursing Association is issuing a leaflet setting forth the objects for which it was formed, the rules of membership, and the advantages obtained by belonging to it. We note that the benevolent side of the association is improving, and there are at present two "Helena" pensions of £20 a year each for aged members who are poor, and that temporary assistance and change to a convalescent home are offered to needy members of not less than two years' training from the benevolent and convalescent funds respectively. It will be remembered that Miss Maud Danks' concert last year raised over £100 for the purpose. A three years' training in a hospital of not less than 40 beds, of which one year must have been spent in a general hospital, is now insisted upon to render candidates eligible.

### RED CROSS NURSES IN THE SOUDAN.

The selection by the Nurses' Co-operation of three nurses for the Soudan has proved an excellent one, and Sir Francis Grenfell's acknowledgment of the value of their services is gratifying not only to themselves and to their co-operation, but to the National Aid Society, under whose auspices they were sent to Egypt.

### A GATHERING OF LEAGUES.

A grand concert in aid of the Catholic Nurses' Institute was held on the 17th inst. at the Victoria Hall, Waterloo Road. There were present representatives of the (Roman) Catholic Leagues of South London, and of the Tower Hamlets, the League of the Cross, the Catholic Social Union, the Society of St. Vincent de Paul, and the Southwark Diocesan Education and Rescue Society, as well as from the public officers in the neighbourhood. An excellent programme was given gratuitously by a large number of well-known artists, and the Rev. Canon Murnane, who had come from Ireland, addressed the assemblage upon the value and necessity of medical advice and skilled nursing in sickness.

### THE PLAGUE IN VIENNA.

The death of Dr. Mueller and one nurse, and the illness of others, from plague in Vienna, has cast a gloom over the city, and alarm and anxiety is universal. Dr. Mueller is said to have been the man most fitted to cope with the plague in Vienna. The nurses have been isolated, and measures have been taken to prevent the further spread of the disease.

### NEW NURSES' HOME AT SUNDERLAND.

Victoria House, an institution opened at Sunderland by the Marchioness of Londonderry last week, is a new nurses' home in connection with the work inaugurated by her ladyship at Stockton. It is affiliated with the Queen Victoria Jubilee Nurses' Institute.

### NURSERY AID SOCIETY.

The Nursery Aid Society is one of the cheapest of nursing associations, as the seven nurses give their services free. During the last four years the Sisters made 3,000 nursing visits in Bristol, and 1,500 in Bedminster. They hold morning training classes at the church house, and distribute food, clothing, and other assistance to the half-starving children attending the dispensary. There are also classes for the instruction of the factory girls, and much good work is done amongst the poor. The expenses for the last four years amounted to £50, exclusive of the stationery bill, which was £59. Thus is much good work done at a very cheap rate. It is cheap because it is unpaid, and thereby hang many considerations. There is a vast amount of unpaid work to be done in the world, and there are also a certain number of independent persons available to do it; but, unfortunately, there are enormous difficulties in setting the workers to the work. First, there is the impossibility of getting volunteers to recognise that unskilled labour is of very little value, and that whatever plough a woman—for the voluntary worker is frequently feminine—puts her hand to, she must learn to drive her furrow straight; next, the age at which women are free to give themselves to good deeds is often when the bloom of first youth is fading and the powers of acquisition waning; then comes the question of discipline, with all the niceties of community life *versus* individual liberty; and, lastly, that lack of enterprise and courage which holds many down to an empty conventional life even while there are careers so rich in usefulness and happiness awaiting them, even though they be most arduous. To countervail the many drawbacks and difficulties that attend the question of free *versus* paid labour, there is one good point. There is no competition for the posts of servitors to the poor; and the bread-winner is not forced to starvation pay by others who have a little money taking less than the living wage.

### WOMEN WORKERS' CONFERENCE.

Mrs Sheepshanks, wife of the Bishop of Norfolk, opened the tenth annual meeting of the National Union of Women Workers, which is held this week at Norwich. Mrs. Alfred Booth is the president, and many well-known leaders among women workers were present. Some of the papers will be valuable to our readers, and therefore we have arranged to be specially represented, and the reports will be published next week.

### A USEFUL RULE.

A useful rule and one that we should like to see universal was rejected by the Guardians of the workhouse, Gravelly Hill, Aston, Birmingham, the other day. One of the members moved that the probationers should not be allowed the use of stimulants excepting with the sanction of the medical officer. He urged very rightly that the measure would be in the interests of the young nurses themselves. The motion was lost, as his colleagues thought that such a regulation would interfere with the liberty of the subject, other officers of the house being free to abstain or not at their own pleasure. Whilst acknowledging that the evil of alcohol lies in the abuse and not in the use of it, there are too many examples of the facility with which the dividing line is passed, for those who have the welfare of the young at heart, not to welcome any reasonable safeguard during the perilous days of early youth before the character is formed. Young people are just as well without stimulants, and if probationers are unable to undergo their training in the heyday of life without them, it is hardly likely that they will be strong enough to stand the stress of a nurse's life in the heat and burden of the day. Preference is often given to abstainers in selecting nurses for important posts, and an early training in the habit of doing without alcohol is in many cases invaluable to their future success. So many nurses fail through intemperance that anything that promises to check the evil should certainly be given a fair trial, and a rule such as the above, while it cannot do any harm and would certainly remove much temptation, will tend to produce a more valuable type of nurse.

### NURSES OF THE LONDON HOSPITAL.

Miss McIntosh, who has been appointed matron of the Government Hospital, Hong Kong, was trained at the London Hospital, and has left to take up her new duties accompanied by Miss Batchelor and Miss Maker (who are appointed "sisters" in the same hospital), as well as by two extra nurses, Miss Robbin and Miss Millington. Miss McIntosh takes the place of Miss Clara Eastmond, also a "London" nurse, who has recently married Dr. J. M Atkinson, an authority on Asiatic diseases. During the summer months the nurses at the "London" have enjoyed many river trips by the kind arrangement of Mr. Hughes, secretary of the Thames Lighterage Company. Parties of night nurses are taken two mornings a week on a steam launch, sometimes up and sometimes down the river, from Blackwall pier, whilst the day nurses have the same delightful outings on two afternoons in every week.

### NURSING LECTURES AT THE HULL ROYAL INFIRMARY.

The medical staff of the Hull Royal Infirmary wil deliver a course of lectures during the winter to the nurses; these will be followed by an examination. The house surgeon will then give a series of lectures on practical work in the wards and the matron another on "Nursing." Arrangements have also been made for a Swedish masseur to hold classes for massage; and for those nurses who can spare the time on the completion of their training at the infirmary to go for either three or six months to a fever hospital.

### SHORT ITEMS.

By a misprint, Miss Ada L. Burgin's term of service as matron of the hospital, Woodstock, South Africa, was stated to be ten years instead of two and a half years.—The Belfast Infirmary nursing staff is to be augmented by five trained nurses and two probationers.—The Bishop of Stepney delivered the address at the harvest festival held at the National Hospital for the Paralysed and Epileptic, Queen's Square, Bloomsbury. The lady superintendent and her assistant decorated the chapel with foliage and flowers from the convalescent home at East Finchley.—Two nurses at St. Thomas's who have had mild attacks of scarlet fever are both doing well.—A new nurses' home is in course of erection at the asylum, Upper Caterham. The many advantages of housing the nursing staffs in separate domiciles are steadily making themselves felt in every department of hospital work.—St. Clement's Maternity Nurses' Home, of which we gave an account last week, is at 87, Fulham Palace Road, and not at Fulham Road, as erroneously given.

### Notes on Gynæcological Nursing.

By ARTHUR E. GILES, M.D., B.Sc., F.R.C.S., Assistant Surgeon Chelsea Hospital for Women.

### V.—NURSING OF PATIENTS AFTER OPERATION.

ONE of the most important parts of the work of the gynæcological nurse is the care of patients after operation. It is also, especially for the first few days, one of the most trying; indeed, I know of nothing that will better test the quality of a nurse, or that will more sharply accentuate the difference between the nurse whose heart is in her work and the one who is nurse in name only. The qualities here required, in addition to the skill and knowledge that come by training, are alert watchfulness, patience, endurance, forgetfulness of self, and conscientious attention to every minute detail. On these characteristics the life of the patient may depend.

During the recovery from anæsthesia a patient is often restless, and shows a tendency to sickness. The nurse's efforts must be directed to keeping her as quiet as possible, and to assisting her if she is sick, so that she may not strain unduly, nor choke. It is best that the patient's head should be turned to one side; a clean towel should be placed under her face to keep the pillow clean, and a small basin should be at hand so that vomited mucus, bile, or other matter may be received in this, and removed from the patient's immediate vicinity during quiet intervals. Some patients are apt to be hysterical at this time, and must be treated as being ill, and not as being troublesome.

After grave operations there is generally more or less shock, indicated by great weakness, a thin quick pulse, coldness of the extremities, and, perhaps, extra restlessness. It is then most important that the patient should be kept warm, not by the heaping up of blankets, but by the application of hot water bottles. In the absence of proper hot-water bottles, an excellent substitute may be found either in bricks placed in the oven and then wrapped round with flannel, or in ordinary claret bottles filled with hot water, well corked, and placed in stockings or covered with flannel. These should be applied to the side of the patient's body, legs, and feet.

Thirst is often a very troublesome feature. If there be a tendency to vomiting it is necessary that nothing should be taken by the mouth, and if the patient can be induced to carry this out for 12 or even 24 hours she will be all the better for it. But if she is much distressed a little ice may be given to suck; or she may have small sips of hot water, or the mouth may be rinsed out with hot or cold water. It is not uncommon, when hot water has been taken in this way, for it to be brought up 10 or 12 hours after operation. If there is no tendency to sickness drinks may be allowed; there is then nothing more refreshing than cold water, and no fear need be entertained about giving it in moderation. Freshly-made tea is also very good, since it is not only refreshing but stimulating. After 12 to 24 hours in serious cases, and much earlier in mild cases, more nourishing drinks may be given, such as milk and water or beef tea. A little milk and soda-water may also be given; but the latter should be in moderate quantity, otherwise the patient may be troubled with flatulence. This leads me to remark that after abdominal operations flatulence may be very distressing; and at first the patient may not be able to get rid of it either by the mouth or by the rectum. Relief is often afforded by a rectal tube, which may be passed in three to six inches, and sometimes even further, but in the latter case the tube should be a fairly pliable one. Failing relief by this means, a small turpentine enema will often act like a charm; half an ounce of turpentine may be given with four ounces of soap and water. In some cases the flatulence is not overcome till the bowels are opened with an aperient and an enema. Great and persistent distension is a bad sign, and it is often one of the characteristics of peritonitis.

I have mentioned two important sources of discomfort after operations, thirst and flatulence; to these must be added a third, namely, back-ache, from lying for some time in the dorsal position. After minor operations there is no necessity at all for this position to be maintained for more than a few hours, whilst after abdominal section I hold the view that patients are kept lying on the back a great deal longer than is necessary. I have known this position to be adopted for a week or ten days, but I believe that before long the practice already adopted by some surgeons, of allowing their patients to be turned on the side as early as the second or third day, will come to be much more widely adopted, Meanwhile this is a matter the responsibility for which does not rest with the nurse. When the dorsal position is necessary, relief from the back-ache may often be obtained by drawing up the patient's knees and placing a pillow under them, or by putting a small flat pillow under the hollow of the back.

A patient should not be left alone at all for 36 or 48 hours after an abdominal section, if it can be avoided; and so, when there is not a night nurse as well as a day nurse, this is a time which may try the nurse's endurance to the utmost. The reason for this necessity is that the patient can do nothing for herself, and every want should be instantly supplied. I have known two cases in which after an abdominal section a patient has been left for a few minutes, and under the influence of great thirst has reached for the hot-water bottle at her feet and drunk some of its contents. Happily they recovered in spite of this escapade, but the risk was a great one. Some patients also will attempt to get out of bed; and it must be remembered that this is a time at which people cannot be expected to act quite rationally. The two risks that attend exertion of this kind are syncope (fainting) from heart-failure, and weakening of the wound. Cases have been recorded in which the abdominal wound has actually given way, and the patient has been found with her intestines lying under the dressings. Needless to say, this is a very serious complication.

I must speak of another point, namely, the use of the slipper and the bed-pan. For the first few days, and indeed as long as the patient is not able to do anything for herself, these should be supplied whenever she asks for them. In private nursing there will probably never be any difficulty about this; but in hospital practice the case is different. It is the rule in some places for the slipper and bed-pan to be supplied at certain fixed times; this may make the nursing rather easier, but it is obviously an irrational practice, besides being a great cruelty to the patients. Docile patients will often put up with much pain and discomfort rather than complain or "give trouble"; other patients become very unruly under these circumstances, and I, for one, should not blame them. A nurse on whom I performed a hysterectomy in hospital told me afterwards that the only feature of the operation that distressed her so much that she would not go through it again on any account was the discomfort she experienced from having the slipper and bed-pan given to her when perhaps she did not require them, and not being able to have them when she really wanted them; and she told me that this was also the experience of the patient lying in the next bed to her. And so I trust that all who have to do with nursing arrangements in hospitals will bear this point in mind; a little extra trouble is of no account compared with this extra burden added to the trials of those who have to undergo serious operations.

I will reserve to the next lecture what I have to say about certain other nursing procedures after operations, and about the indications that may be afforded that complications to convalescence are threatening.

### Training in the Provinces.

(Continued from page 39.)

### ROYAL SOUTHERN HOSPITAL, LIVERPOOL
(200 Beds).

### Terms of Training.

Applicants for training at the Liverpool Royal Southern Hospital must be between 21 and 34 years of age. After the usual month's trial, if a candidate proves eligible, she is accepted for a term of two years' training, and expected to remain in the service of the hospital for four years, with the chance of promotion at the end of two to the position of staff nurse, and at the end of the fourth year to that of sister. No salary is given during the first year, but commences at £20 the second year, rising gradually to £25 when appointed on the staff. Sisters' salaries range from £25 to £40. Laundry and indoor uniform are provided by the hospital for all the nursing staff.

A certain number of paying probationers are received for training at a payment of £1 1s. per week.

Lectures are given to the nurses during their training by members of the honorary staff, and classes are held by the home sister, certificates being granted after passing examination and completing the engagement. Teaching in sick-room cookery is given. There is a private nursing staff attached to the hospital, on which nurses may be appointed after completing two years' training in the wards. Staff nurses (two to each ward) take night and day duty alternately, two months at a time.

### Hours On and Off Duty.

The hours on duty for day nurses are from 7.30 a.m. to 9 p.m., with time for meals, and two hours daily for recreation. Four hours' leave is given twice a week, and one day each month, on which nurses do not go on duty at all in the wards. The sisters do not go on duty in the morning till 8.30. Yearly holiday is, for probationers, two weeks; for staff nurses, three weeks; and for sisters, four weeks.

### Meals.

All meals are served in the nurses' dining-room, and no weekly "food allowances" are made to nurses on day duty. Night nurses have tea, sugar, and butter served out to them for their meal during the night. The day nurses breakfast at 7 a.m.; dinner is served at 11.30 a.m.; tea from 3.30 to 4.30; and supper at 9. Sisters' breakfast is at 7.30 a.m.; dinner at 12.30, tea from 5.30 to 6.30, and supper at 9.

### LIVERPOOL ROYAL INFIRMARY (341 beds).

### Terms of Training.

The Liverpool Royal Infirmary is nursed by the Liverpool Training School and Home for Nurses, probationers being bound to the service of the institution for four years, three in training in the hospital wards, and for a fourth as district or private nurses. Candidates should be well-educated and intelligent women, between 25 and 35 years of age. If accepted at the end of the trial month an agreement is signed, and salary begins at once at £10 for the first year, increasing to £16 for the second year, and to £18 for the third. £25 is paid for the fourth year. Sisters' salaries range from £30 to £35 per annum. Full uniform and laundry are provided for all the staff. Probationers attend lectures given by members of the medical and surgical staff, and classes are held by the assistant lady superintendent at the home, all probationers and nurses being required to pass the prescribed examinations. Certificates are given at the end of the four years, and may be subsequently extended if the nurse remains in the service of the hospital. These certificates may, however, be cancelled on the register for subsequent misconduct. A certain number of "special probationers" are received for one year's training in hospital and district nursing, and are subject to the same conditions as ordinary probationers in relation to their work, &c.; they pay £5 upon entering for the trial month, which sum is not returnable; £30, including this deposit, payable in advance, for the first six months' training; and £20 for the second six months. These payments include board, lodging, laundry, and indoor uniform. If considered suitable special probationers may afterwards extend their engagement for a further period of two years, when they receive salary on the same terms as ordinary probationers, and £15 of the premium is returned to them. No certificates are granted under the full term of three years' training. A pension of £20 per annum is granted to nurses after twenty years' service.

Night duty is taken for three months at a time, alternately with day duty, by nurses in their second and third years. No night duty is required of first year probationers.

### Hours On and Off Duty.

The working day at the Liverpool Royal Infirmary is from 7 a.m. to 9 p.m.; during these hours two hours off are allowed every day, either in the morning or afternoon, and half an hour each is given for lunch, dinner, tea, and supper, so that the actual hours on duty in the wards number about ten. On alternate Sundays probationers have leave of absence from 5 till 8.30 p.m., and a whole day off once a month.

Staff nurses are off duty on alternate days from 2 to 4 p.m., and from 5 to 8.30 p.m.; on alternate Saturdays from 2 to 9 p.m., alternate Sundays from 5 to 9 p.m., and once a month for a whole day. The sisters are on duty in their wards between 8 a.m. and 9.30 p.m., taking their time for recreation on alternate days from 2 to 4 p.m., or from 5 to 8.45 p.m. Time for meals has also to be deducted from these hours. On alternate Saturdays sisters are off duty from 2 to 9 p.m., and on alternate Sundays from 5 till 9 p.m. Once a month they have leave from 2 p.m. on Saturday to 9.30 p.m. on Sunday. Night nurses are on duty from 9 p.m. to 8.15 a.m., taking their recreation between 10 and 12.30. One day off duty is given for every month of night duty, the days off being taken at the end of a term of night work. Annual holidays are three weeks for probationers and staff nurses, and four weeks for sisters.

### Meals.

All meals are served in the nurses' home, where all the cooking for the nursing staff is done. Special attention is paid to the dietary, with the object of giving the nurses as much variety as possible in their food. Probationers and staff nurses have breakfast at 6.30 a.m., lunch is from 9 to 9.30, dinners between 11.30 and 12.30, tea from 4 to 5, and supper from 8 to 9. The sisters breakfast at 7.30 a.m., dinner is served at 11.30, tea they have in their own sitting-rooms, and supper is at 8 p.m. Night nurses have their breakfast at 8.30 p.m., and dinner at 9 a.m.

### Minor Appointments.

Walsall District Hospital.—Miss Kate Hunt has been appointed Night Sister here. She was trained at Bristol General Hospital for three years, where she gained the first prize for anatomy and physiology, and the first prize for medical and surgical nursing in the junior nurses' examination in 1896, also in the senior nurses' examination in the following year she was awarded the first prizes for physiology and for medical and surgical nursing. She took the L.O.S. certificate last April.

Wolverhampton Workhouse Infirmary. — Miss Alice Maria Williams, who was trained at the Wolverhampton Union Infirmary, was appointed Charge Nurse of the above infirmary on the 7th inst.

Union Infirmary, Grimsby.—Miss Kate L. Hodges, who has just completed her three years' training at the Union Infirmary, Sunderland, has been appointed nurse of the above. She holds the certificate of the L.O.S.

### Nursing in Paris Hospitals.

### C.—THE NURSING SISTERS.

### VI.—Professional Estimation.

Hospital doctors, like other public officials, are apt to condone or support existing governmental arrangements, unless it be the inadequate remuneration or appreciation of their own services by an ungrateful country. Nevertheless, in their heart of hearts, I believe most of the doctors are thoroughly distrustful of the possibility of the new laic filling in all respects satisfactorily the functions performed by the Sisters. This distrust was well evidenced by the very strong protest against the laicisation sent by almost the entire medical and surgical staff of the various hospitals to M. Allain Targé, the Minister of; the Interior, on November 17th, 1885.

In the second article of Series A I promised to again refer to the protest. It is so important as evidence of the professional opinion on the Sisters when they were still in office, or only recently evicted, and when it was given against the well-known desires and intentions of the administration employing these professional experts, that I think it worth while to give the list of the signatories. More than one of the names is of world-wide fame in some special line of pathology or surgery, and, of course, many of them have since passed over to the majority.

The protest, after stating that "the undersigned doctors and surgeons of the hospitals of Paris have the honour to ask you (*i.e.*, M. Allain Targé, the Minister) for the maintenance of the nuns in the hospital services to which they are attached," and that, "in making this demand, they consider that they are acting in the interests of the patients as well as for the good order and conduct of the hospitals and hospices of Paris," is signed in regular order as follows: Dujardin-Beaumetz, Gouraud, doctors, Marchand, surgeon, of Cochin Hospital; Potain, Blachez, Rendu, Rigal, doctors, Gwyon, Le Fort, surgeons, of Necker; Hérard, Moissenet, Marotte, Nonat, Bouchwt, Hervieux, Barthez, Gueneau, de Mussy, Bergeron (President of the Academy of Medicine), Marjolin, honorary physicians of the Hôtel Dieu and Children's Hospital; Gosselin (member of the Institute), Ricord, A. Guerin, Monod, Cusco, Desormeaux, honorary surgeons of the Hôtel Dieu, La Charité, &c; Moutard, Martin, Empis, Bucquoy, Vulpian (member of the Institute), doctors of the Hôtel Dieu; Richet, Panis, Tillaux, surgeons at Hôtel Dieu; Hardy, Laboulbne, Peter Téréol, Luys, Desnos, doctors at La Charité; A. Desprès, surgeon at La Charité; Mesnet, Hayem, Dieulafoy, Tennesson, Landrieux, doctors at Saint Antoine (hospital already laicised); Delens, surgeon at Saint Antoine; Lecorché, E. Labbé, doctors, Horteloup, Marc Sée, surgeons at the Maison de Santé; A. Fournier, E. Besnier, doctors, Péan, Le Dentu, surgeons at Saint Louis; Millard, Gombault, Fernet, J. Guyot, doctors, L. Labbé, Cruveilhier, surgeons, at Beaujon; Demontpallier, doctor, Polailton, surgeon, at La Pitié (hospital already laicised); Danlcs, R. Moutard - Martin, doctors, Gilette, surgeon, at Tenon (hospital already laicised); Triboulet, D'Heilly, Cadet de Gassicourt, doctors, Lannelongue, surgeon, at Trousseau; Labric, J. Simon, Descroizelles, A. Ollivier, doctors, De Saint-German, surgeon, at Hôpital des Enfants; Sevestre, doctor, Guéniot, surgeon, at Enfants Assistés; Constantine Paul, doctor at Lariboisière; Du Castel, doctor, Humbert, surgeon, at Midi Hospital (already laicised); Lebadie-Lagrave, doctors at Maternité Hospital (laic hospital); Rocques, Martineau, doctors at Lourcine (laicised); Goughenheim Huchard, doctors at Bichat (laic hospital); Berger, surgeon at Bicêtre (laic hospital); Ferrand, doctor at Laënnec (laic hospital); Gingeot, doctor at Sainte Périne (laic hospital); Balzar, Barrié, Merklen, Chauffard, A. Robin, Renault, Mwzelier, Moizard, Barth, Oulmont, Brocq, doctors, Jalaguier, Nélaton, Quenú, Bouilly, Félizet, Schwartz, Routier, Second-Brun, honorary surgeons of the hospitals of Paris. Doctors Guibout (of Saint Louis) and Pozzi (of Lourcine) concurred in the petition.

The reply of the "apostle of laicisation" to this serious challenge was that only 55 out of 155 doctors and surgeons in active service had agreed. From this Dr. Bourneville assumed that the round hundred remaining were all in favour of the arbitrary proceedings of himself and his supporters. This is rather a high-handed deduction, it seems to me. The doctor was however very nettled, and in a long and very clever editorial article in his "Progrés Médical" (March 19th, 1881) combated the famous letter with more tenable arguments. Beginning with the remark that it could not be considered a religious controversy when Israelites, Protestants, and Freethinkers were found among the demandants for the retention of Catholic nuns, Dr. Bourneville argued that the letter was really a demand for the eviction of laic matrons, &c., in hospitals where the Sisters had never had charge. Moreover, he pertinently inquired how the chiefs of six hospitals who had never had the Sisters under them, yet who had signed the letter, could claim to have experience in the matter? These points and some others are certainly of apparent importance; but it can be replied that in drafting a general letter for all the hospitals, with their varied history and installations, no form of statement could well be made to exactly fit all the representative officials.

In the same article Dr. Bourneville deals with a letter addressed on March 8th (two days before the general letter) to the directors of the Assistance Publique by the medical staff of the New Tenon Hospital, and protesting in much stronger terms. The doctor's reply is quite naturally in a more violent key. As has unfortunately been too much the case with him and his fellow spirits for over twenty years past, gross insults to the Sisters are flung about, but there is an important statement of the programme in reference to "what is to replace them." This paragraph is the gist of the case for the whole laicising campaign. He says: "What will replace the Sisters? We expect it will be women who have passed all the stages of the hospitaliary hierarchy, having followed the special and professional courses, those, for instance, which our friends have so devotedly founded at La Salpetrière and Bicêtre. They will have, beside the ordinary and current education, practice in wound dressing and sufficient knowledge of the usual medical terms, so as not to sort pills into black, white, little, and big, and bottles into blue and green, as do so many of the nuns. They will understand that in a hospital the principal person is the head doctor, and that he must have all information of the day and night at his morning round. They will learn that the patients are human beings, not condemned sinners, that in a hospital we nurse only the body, and that liberty of conscience must be respected. In brief, they will know how to nurse the sick, for they will have become matron nurses after having been for a long time under other matrons and the doctors. On the contrary, What do the Sisters know? Nothing beyond the education received in their families. Nothing outside of their religious duties. Nothing, not one thing the Mother Superior does not wish to let them know. The novices disappear like white doves when the morning round begins: they are concealed lest these recluses should be exposed to the world. Where then do they learn how to nurse the sick? Is it in giving out food or in shrouding the dead, the only tasks they are willing to assist in?"

This is a most ferocious indictment, and from a very prejudiced if professional source. That a large number of doctors who come in contact with the Sisters in daily work were of different opinion is evident in a general way by the famous round robin, and in a particular manner by the utterances of various individuals. Thus Dr. Gringeot, of St. Antoine Hospital, in a published plea for the Sisters set down four special points in their favour: (1) That the Sisters having little connection with the outer world, rarely leave the hospital, something so frequent with laics, to the injury of the service; (2) that the Sisters rarely have vacations, the laics always; (3) that the Sisters have no objection to being attached all their lives to the same hospital, and even to the same ward, something the laics would never consent to, and which Dr. Gringeot thinks a great advantage; (4) that the Sisters, as celibates, have no distracting calls of other social relations. Again, Dr. Ferraud, of the Lourcine hospital, instanced as an absolute proof of the superiority of the Sisters as matrons over the new laics that the understaff had to be increased right after the change, and gave three chief points against the laics as compared with the Sisters: (1) Lack of control over both patients and under nurses; (2) faulty reports of what passes in the wards; (3) frequent change of personnel.

But the pros and cons of the professional opinion have been almost as varied in character as there are doctors to give them. Truly it has been a case of "Where doctors disagree," and it rests for the laity after all to decide. EDMUND R. SPEARMAN.

### Royal British Nurses' Association.

A GENERAL Council meeting of the Royal British Nursing Association was held at 11, Chandos Street, W., at five p.m. on October 20th, 1898. Sir JAMES CRICHTON BROWNE took the chair. Two reports, one from the Hon. Treasurer, Mr. Langton, and one from the Executive Committee, were presented and passed, and two vacancies on the General Council were filled up by the appointment of Miss Amy Catherine Robinson and Miss Ainsworth.

The financial report for the three months ending September, 1898, showed an expenditure of £207; some of the items, however, were extraordinary, as was, for instance, the printing in connection with the promulgation of the new bye-laws. It is hoped that the *Nurses' Journal* will shortly bring in a revenue of about £30 a year, instead of causing a deficit as hitherto. Of the balance at the bank on the 1st inst., £162 has been appropriated for the Benevolent Fund and the Investment Fund.

Mr. LANGTON, after reading the report, drew attention to the need of having the finances on a firmer basis, and urged on the members their duty towards the Association of paying their own subscriptions, and also of inducing other qualified nurses to join it. The committee would have to be very careful, if not something more, as regards expenditure, and the outside public should take an interest in it. Mr. Peter Reid had sent a donation of £10 by Sir James Crichton Browne, who himself gave £3. With regard to the proposal for life members to repay their subscriptions, Miss de Pledge, on behalf of several friends, said that she and they were willing to do so, but that before any steps were taken in the matter they would like Dr. Bezly Thorne's services to the Association recognised in a substantial manner. The Chairman said that this suggestion should be brought forward and considered in due time.

Mr. FARDON then read the report of the Executive Committee, which showed that 43 nurses had been registered during the last six months, 47 members had been elected, 30 (half of whom are in arrears with their subscriptions) had withdrawn, and 2 had died. Up to the present time the following ladies and gentlemen have accepted *ex-officio* seats on the council: Sir Dyce Duckworth, M.D.; Sir Edwin Saunders, F.R.C.S.; Hector Cameron, M.D., president of the Faculty of Physicians and Surgeons, Glasgow; F. J. Gant, F.R.C.S.; Professor Latham, M.D.; F. W. Pavy, M.D., F.R.S.; Miss Hogg, head sister, Royal Naval Hospital, Haslar; Miss M. E. Jones, matron, the General Infirmary, Birmingham; Miss Loch, senior lady superintendent, I.A.N.S.; Miss Lofts, matron, the Lewisham Infirmary; Miss Medill, lady superintendent, St. Mary's Hospital, Paddington; Miss de Pledge, matron, the Chelsea Infirmary; Miss Elma Smith, matron, the Central London Sick Asylum; Mrs. Strong, matron, the Royal Infirmary, Glasgow; Miss Thorold, matron, the Middlesex Hospital; Miss Wedgwood, matron, the Royal Free Hospital; Miss Wesley, matron, St. George's-in-the-East Infirmary.

Remarking upon the report, Mr. FARDON said that all the members ought to realise their responsibilities towards the Association, and work together for and with it. In the matter of paying their subscriptions regularly, for instance, there were many who availed themselves of the privileges of the Association but were more or less in arrears. This ought not to be.

After some discussion, Sir JAMES CRICHTON BROWNE said that arrears in subscriptions could be recovered in a court of law, and that it was possible to refuse a resignation if the subscriptions had not been paid up to date. Proceedings to recover the debt might therefore be taken against the resigning members who had not yet paid in full.

There were present, beside the Chairman, the Hon. Treasurer and Hon. Medical Secretary, Miss Thorold, Mr. F. J. Gant, F.R.C.S. (who seconded the financial report), Dr. Alderson (who supported the same), Dr. Wethered (who seconded the report of the Executive Committee), Dr. Bezly Thorne, Miss de Pledge, Miss Foggo Thomson, Miss Meyrick, &c.

In the new leaflet setting forth the object and advantages of the R.B.N.A., attention is called to the fact that after a candidate has been registered as a nurse she has still to be elected a member of the Association, and all nurses accepted for registration are cordially invited to apply at once for election on the form sent for that purpose.

### The Army Nursing Sister.

By a Correspondent.

The facts relating to the position, duties, pay, and pension of the Army Nursing Sister are known to very few beyond the comparatively small number whom they concern, and the outside public are consequently under much misapprehension upon these subjects.

There are in the Army some 72 sisters who are never allowed to go farther East than Aden. It is understood that they are ladies by birth and education, are trained nurses, and at date of entry under 35 years of age. For the last few years it has been necessary that they should hold a three years' certificate. Army nursing is under the direction of the medical officers, and is carried out by the ward masters (themselves trained nurses, and usually holding a certificate qualifying them to dispense), assisted by trained orderlies; the position of the nursing sister is therefore an anomalous one. She does not directly rule the ward—she supervises, and her appointment is from the War Office.

Her duties are to teach the orderlies all she can of surgical and medical nursing, and sometimes massage; to inspect and see that the patients have what is needed, that they are kept clean and properly fed, to give medicines, to carry out the medical officers' directions, and to report any neglect of duty on the part of the orderlies. A sister's success, therefore, depends upon her recognising her position, and the fact that she is dealing with and teaching men over whom she has but limited control, and not with probationers. Still, a sister, by using tact and taking trouble, can get all that is necessary for the sick under her supervision. The soldiers are well nursed. The ward masters are, as before noted, trained nurses; and, in addition, trustworthy men. They are either corporals or sergeants specially selected for these positions. They are responsible for the equipment and cleanliness of the wards. The orderlies have periodical lectures and demonstrations from their medical officers as well as the teaching in wards.

The sisters are on duty all day, and, in event of necessity, until a late hour at night, and there is always an orderly medical officer who can be called at a moment's notice. The pay of the sister is fair. It begins at the rate of £30 a year, with uniform and allowances, and rises £2 annually to £50. Many improvements have been introduced within the last few years; for instance, the maintenance allowances are continued through leave. But the pensions are inadequate, and the present system of special pensions incongruous. Take the following incidents as illustrations: One sister, invalided after 11 years' service, receives a pension of £14 19s. 3d. a year; another, 60 years of age, after 22 years' service, gets £36; whilst another, who has only the Netley training, and who has enjoyed delicate health and an abundance of sick leave for 14 years, is awarded a special pension of £40 a year. All these pensions, the special one included, are absurdly small. No educated woman can live really comfortably under 25s. a week, especially when age and delicacy are factors in the matter, and the question to consider is what portion of this cost ought to be borne by the State.

With regard to the advantages and disadvantages of male versus female nursing, there is no doubt that nursing is more a woman's work than a man's; but after all is said, that can be said on both sides, the fact remains that all depends upon the individual nurse, and upon the motive that actuates his or her conduct. The heart and thoughts must be with the sick, and not in the distractions of social pleasure, to ensure a high standard of nursing anywhere.

There is much written about the value of the nurses supplied by the various Red Cross Societies, and Army medical authorities have been somewhat severely criticised for their reluctance in accepting their co-operation, but it must not be forgotten that the most excellent civil nurse has much to learn of Army technicalities before her full value can be felt.

There is some doubt if the present number of Army nurses is sufficient even in times of peace, but it is certainly far from adequate in war, or for epidemics such as of enteric fever or diphtheria.

### Zenana Bible and Medical Mission.

A valedictory meeting of the Zenana Bible and Medical Mission to the outgoing missionaries was held in Exeter Hall on Thursday, the 20th inst., at three p.m. Mr. T. A. Denny took the chair, and there were present, besides the ladies leaving for India either for the first time or after furlough, Lord Kinnaird and the Hon. Gertrude Kinnaird; the Rev. T. S. Webster, M.A., who gave the address; the Rev. A. H. Wright; and the Rev. A. H. Bowman. The hall was well filled, and each of those leaving to resume work in India gave a short farewell address. One or two points were brought into prominence. The comparatively small results obtained were excused by describing the magnitude of the fabric of superstition to be overthrown, and by the hardships to be faced by converts professing Christianity. The efforts made to preach the Gospel were justified by telling something of the magnitude of the work, the greatness of the need. "If you have known these glad tidings so long, why did you not come and tell them to us sooner?" was a perfectly fair inquiry made by some of the hearers, and one difficult to answer in face of the command, "Go and preach the Gospel." One lady drew attention to the fact that the drink bill for Great Britain for three days amounted to a larger sum than was spent on all foreign missions in a twelvemonth. The proportion of women workers at home is one in every 50 women. In India it is one in 100,000.

### St. John's House.

The committee of St. John's House, in Norfolk Street, show themselves anxious to promote the welfare and comfort of the nurses on their staff. It has just been resolved for the future to take a quarter-share of the profits of each year and divide it in bonuses amongst those nurses who have been at least four years working for them. The other three-quarters will be invested, as hitherto, in the pension fund in connection with the House. The first division will be declared in the new year, and will be based on the report of the auditors for 1898. The pensions attached to this society vary from £15 a year to £26. The former is granted to nurses disabled by delicate health for work after 12 years' service; the latter to nurses who have served the society for 26 years and upwards. Nurses of from 20 to 26 years' service are pensioned at the rate of £1 for every year.

### Appointments.

Belfast Hospital for Sick Children.—Miss Amy Mactaggart, who was trained at the Westminster Hospital, was, on October 19th, appointed Lady Superintendent here. Miss Mactaggart has been surgical and theatre sister at the Royal Hospital for Children, Edinburgh, and afterwards sister and matron's assistant at the Westminster Hospital.

Poole Union Workhouse.—On the 12th inst. Miss Jessie Hodges was elected Superintendent Nurse of this workhouse. She was trained at the Public Hospital and Dispensary, now known as the Royal Hospital, and has held the posts of assistant nurse at the Bristol Royal Infirmary and at Barton Regis Union Hospital. She holds the certificate of the L.O.S.

Fife and Kinross District Asylum.—Miss Phœbe Berwick has been appointed Matron of this institution. Miss Berwick was trained at the Edinburgh Royal Infirmary, and subsequently acted for two years as staff nurse at the Perth District Asylum, Murthly.

### Presentations.

Miss Copeman, on the resignation of her post as Superintendent of district nurses, which she had held for eight years, in Sister Katherine's Home at Plaistow, was presented with a handsomely fitted nurse's bag by her friends and nurses. Miss Copeman is taking up nursing work in Oxford.

### For Reading to the Sick.

### THE BETTER LAND.

### Verses.

I AM hast'ning homeward, to the land I love,
Would'st thou bid me linger, from those realms above?
Soon I'll be with Jesus! see Him face to face!
Then I'll sing the glory of His wondrous grace!

In His presence standing, I my voice shall raise
In a sinless anthem of eternal praise;
Praise to Him who brought me from darkness into light,
Put away transgression, clothed my soul in white.

Here I have had my sorrow; there, shall be no more;
Hushed is every wavelet on yon glorious shore.
One by one they're gathering home, from every land;
Soon I'll pass the desert—join their happy band.

Why, then, should I murmur? though the way be rough,
Jesus, He will guide me, is not that enough?
Then let clouds o'ershade me, still I need not fear,
His strength shall sustain me, His sweet voice shall cheer.

Brief life is here our portion,
Brief sorrow, short-lived care;
The life that knows no ending,
The tearless life is there.

And now we fight the battle,
And then shall wear the crown
Of full and everlasting
And passionless renown.

The morning shall awaken,
The shadows shall decay,
And each true-hearted servant
Shall shine as doth the day.

Then God, our King and Portion,
In fulness of His grace,
Shall we behold for ever,
And worship face to face.

Who follows in His train?
Who best can drink His cup of woe,
Triumphant over pain;
Who patient bears His cross below,
He follows in His train. —*Heber*.

### Reading.

"The inhabitants shall not say, I am sick."—*Isaiah* xxxiii. 24.

These words carry the mind at once to the New Jerusalem, "which cometh down from God out of heaven prepared as a bride adorned for her husband." Our blessed Lord was an inhabitant of it originally. He never said, I am sick.

What a world it must be! There must be no elements of sickness therein. . . . The sun will not smite by day nor the moon by night. There will be "no more sea"; no "fulness of bread"; no "excess of wine"; no "midnight cry." The water will be "pure as crystal." The fruit will grow on a "tree of life." . . . The blind will not cry out by the roadside. What a world must be that of which such things are truly spoken! . . .

If we would enter the kingdom of Heaven we must be "converted, and become as little children." Conversion is not a barrier to keep us out, but a "door of hope" to let us in. How else could the lost sheep re-enter the fold, the ten virgins rekindle their lamps, the prodigal son regain his Father's house—how could any who have "erred and strayed" fall at Christ's feet, cling to His cross, and obtain "the remission of sins that are past through the forbearance of God"? Men shrink oftentimes from the word "conversion" as though it involved a "hard saying"; whereas it is of all other words the most precious. It is a passport to that city where the inhabitant never says, I am sick.—*Rev. J. Bateman*.

### Wants and Workers.

THE District Nurse, Castle View, Clitheroe, Lancashire, would be glad to know of anyone having no further use for a Merlin or other self-propelling chair, suitable for a heavy woman, who would kindly give (or sell it cheaply) to a workman's wife crippled by chronic rheumatism.

### Notes and Queries.

**The contents of the Editor's Letter-box have now reached such unwieldy proportions that it has become necessary to establish a hard and fast rule regarding Answers to Correspondents. In future, all questions requiring replies will continue to be answered in this column without any fee. If an answer is required by letter, a fee of half-a-crown must be enclosed with the note containing the enquiry. We are always pleased to help our numerous correspondents to the fullest extent, and we can trust them to sympathise in the overwhelming amount of writing which makes the new rules a necessity.**

**Every communication must be accompanied by the writer's name and address, otherwise it will receive no attention.**

*Qualified Dispenser.*

(46) Would you kindly let me know what steps I ought to take to become a qualified dispenser, and what books I would require to study for the examination?—*E. E.*

See in "Mirror" for April 3rd, 1897, "How to Become a Dispenser.'

*Completion of Training.*

(47) I have had two years' training in a provincial general hospital. Some time ago I left in order to accept a post abroad that had been offered me. Circumstances have arisen to prevent my going. Is it possible for me now to gain a three-years' certificate by going to some other general hospital for one year? I thought I saw a paragraph in your paper some time ago stating that some hospital in London had made arrangements whereby nurses could do so.—*H. B.*

The Hospital for Diseases of the Chest, Victoria Park, E. We should recommend your endeavouring to re-enter your first training school if possible.

*Books for Probationers.*

(48) Can you inform me what books are generally used in a workhouse infirmary for lecturing to pro's in their first, second, and third year?—*Matron.*

There is no one book generally used. We send you a list of books published by the Scientific Press.

*A Question of Title.*

(49) Has a nurse who holds a certificate for monthly nursing and L.O.S. diploma any right to call herself a trained nurse? No other training.—*H. B.*

Certainly not. She is a midwife, and has not been trained in general nursing.

*A Year's Training.*

(50) Can you tell me of a hospital where I can get one year's training? —*L. F.*

See our advertisement columns.

*Clear Starching.*

(51) Can you give me any information on the subject of washing and polishing my own collars and cuffs?—*District Nurse.*

Plunge them in lukewarm water and wash. Soap well, and boil them for 15 minutes. Rinse them well. Prepare cold starch and the "gloss" in accordance with directions given on packets, and rub the linen thoroughly in the mixture. Stretch them flat on a clean towel, and with a piece of soaped rag take off every bit of outside starch. Roll tightly in towel and let them lie for 24 hours. Iron on a clean ironing sheet with a hot iron until dry, the wrong side first. When ironing at the right side move the iron swiftly to polish. Laundresses use a special iron for the last process, and experience is needed to acquire the knack.

*Male Nurses.*

(52) Will you kindly inform me where in England and on the Continent male nurses are trained?—*Lynton.*

Nurses are trained in England at the National Hospital for the Paralysed and Epileptic, Queen's Square, Bloomsbury, W.C., and in the larger lunatic asylums. There are several schools in America. The New York City Training School, Blackwell's Island, offers a good course of instruction.

*Nurses Abroad.*

(53) Can you tell me of any nursing institute which sends nurses abroad, or any other way for private nurses to go abroad?—*C.*

The Hollond Institute, 1, Tavistock Chambers, Bloomsbury, W.C. You might advertise your willingness to accompany a patient, in which case you must carefully inquire into references.

*Notice.*

(54) Is it possible for a nurse to cancel an engagement? 2. How long notice should she give if her fees are charged by the week? 3. Can a patient discharge a nurse without notice or compensation?—*E. M. E.*

1. A nurse cannot cancel an engagement; if not released by her employer she must fulfil it or provide a substitute. If she does neither the one nor the other the patient can engage a substitute and the nurse is liable for any excess of fees. 2. If paid by the week a week's notice should be given. 3. It entirely depends on the terms of agreement.

*Nurses for the Riviera.*

(55) Kindly tell me the address of the Society for Providing Nurses for the Riviera?—*E. M. T.*

See next query.

*Nursing Abroad.*

(56) Could you tell me how to proceed in order to get into some hospital or nursing home abroad, France preferred?—*E. M. H.*

See advertisements, and write to Director, The Hollond Institute, 1, Tavistock Chambers, Bloomsbury, S.W.

*Lady Roberts' Nurses.*

(57) I am anxious to go out to India as one of Lady Roberts' nurses, and should be glad if you can give me any information on the subject.—*K. S.*

Lady Roberts chooses her own nurses from personal acquaintance. She never considers applications from others.

